# Comparisons and correlations of pain intensity and respiratory and peripheral muscle strength in the pre- and postoperative periods of cardiac surgery

**DOI:** 10.5935/0103-507X.20180069

**Published:** 2018

**Authors:** Thayse Campos de Menezes, Daniela Bassi, Ricardo César Cavalcanti, Juliana Emanuelle Santos Luz Barros, Karolyne Soares Barbosa Granja, Ana Carolina do Nascimento Calles, Ana Luiza Exel

**Affiliations:** 1 Departamento de Fisioterapia, Centro Universitário Tiradentes - Maceió (AL), Brasil.; 2 Departamento de Fisioterapia, Universidade Ceuma - São Luís (MA), Brasil.; 3 Centro de Pesquisa Clínica em Cardiologia, Hospital do Coração - Maceió (AL), Brasil.

**Keywords:** Rehabilitation, Pain, Cardiac surgery, Respiratory muscles, Muscle strength, Postoperative period

## Abstract

**Objective:**

To evaluate respiratory and peripheral muscle strength after cardiac surgery. Additionally, we compared the changes in these variables on the third and sixth postoperative days.

**Methods:**

Forty-six patients were recruited, including 17 women and 29 men, with a mean age of 60.50 years (SD = 9.20). Myocardial revascularization surgery was performed in 36 patients, replacement of the aortic valve in 5 patients, and replacement of the mitral valve in 5 patients.

**Results:**

A significant reduction in respiratory and peripheral muscle strength and a significant increase in pain intensity were observed on the third and sixth postoperative days (p < 0.05), except for the variable maximal inspiratory pressure; on the sixth postoperative day, maximal inspiratory pressure values were already similar to the preoperative and predicted values (p > 0.05). There was an association between peripheral muscle strength, specifically between maximal expiratory pressure preoperatively (rs = 0.383; p = 0.009), on the third postoperative day (rs = 0.468; p = 0.001) and on the sixth postoperative day (rs = 0.311; p = 0.037). The effect sizes were consistently moderate-to-large for respiratory muscle strength, the Medical Research Council scale and the visual analog scale, in particular between preoperative assessment and the sixth postoperative day.

**Conclusion:**

There is a decrease in respiratory and peripheral muscle strength after cardiac surgery. In addition, maximal expiratory pressure is the variable that is most associated with peripheral muscle strength. These variables, especially respiratory and peripheral muscle strength, should be considered by professionals working in the intensive care setting.

## INTRODUCTION

Cardiac surgeries are still considered the procedures of choice for reducing symptoms and mortality.^(1-3)^ The main cardiac surgeries are myocardial revascularization surgery (MRS), surgery for valvulopathies, correction of aortic diseases, and cardiac transplantation.^(1,4)^

In this context, surgical stress induces loss of muscle mass due to dysregulation in protein metabolism.^(5,6)^ This condition culminates in the reduction of muscle strength, causing long-term deficiencies such as persistent muscle weakness.^7^ Therefore, prevention of muscle proteolysis induced by surgical stress in the early postoperative phase may be a potential intervention for preserving skeletal muscle strength after cardiac surgery.^(8)^

Chest opening during cardiac surgery may affect nerves and respiratory muscles, but it is not yet clear in the literature whether decreased respiratory muscle strength (RMS) would be a possible cause of respiratory compromise in these patients. In addition, decreased preoperative RMS has been shown to prolong mechanical ventilation in the postoperative (PO) period and is described as a determinant of decreased functional capacity.^(9)^

In cardiac surgery patients, decreased RMS has been associated with decreased functional capacity and has contributed to a prolonged period of recovery of lung function and the occurrence of physical deconditioning, which can last for several weeks.^(10)^ Respiratory repercussions also generate changes in RMS, as well as changes in lung volumes and capacities, alveolar dysfunction, depression of central respiratory stimulation, and mechanical disorders of thoracic function.^(3,4,11)^ In addition, it is known that most cardiac surgery patients present with episodes of muscle weakness in the preoperative period, which is accentuated after the surgical procedure.^(12)^ However, this muscle weakness is more noticeable in the respiratory muscles than in the peripheral muscles, although the latter muscles are also inactive.^(12,13)^

Another important factor in this context is the role of postoperative pain in the functional recovery of the patient, which is an important indicator to estimate the physical and psychological tolls because prolonged painful stimuli cause suffering and complications in the PO period, which correlate with increased morbidity and mortality by affecting the ability to cough, breathe, and move properly.^(14,15)^

In view of the above, the objectives of this study were to evaluate RMS and peripheral muscle strength (PMS) after cardiac surgery and to analyze the changes in these variables on the third and sixth PO days, observing possible alterations in maximal respiratory pressures and possible correlations with PMS and pain.

## METHODS

This was a longitudinal observational study. The data collection was carried out from March to October 2016 after approval of the project by the Research Ethics Committee of the *Centro Universitário Tiradentes* (protocol number 40004314.6.0000.5641). The nonprobabilistic convenience sample was composed of patients in the pre- and PO periods for cardiac surgery who were admitted to *Hospital do Coração* of Alagoas (Maceió, AL, Brazil). In compliance with Brazilian and international ethical standards, patients were informed about the procedures to be performed and signed an informed consent form to participate in this study.

Patients admitted to the hospital, both men and women over 18 years of age, underwent MRS and midsternotomy for valvular changes. Of these, patients who were cognitively impaired were excluded from the assessment of RMS, PMS, and pain intensity. Patients who had hospitalization-related complications that prevented their reevaluation were also excluded.

The evaluations were carried out at three time points. In the preoperative period (on the day before cardiac surgery), an evaluation form with identification, disease, treatment, and anthropometric data was used; RMS, PMS, and pain intensity were measured.

**Figure 1:**
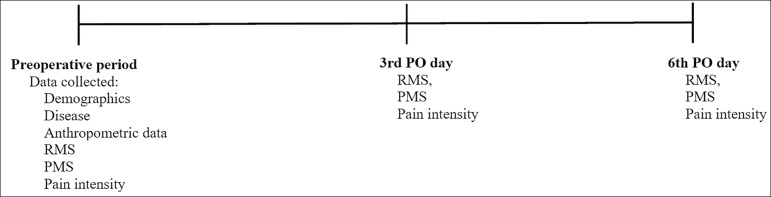
Timeline of the evaluations.

On the third and sixth PO day, the same evaluations performed in the preoperative period were performed again by the same evaluator as shown in the figure 1. The patients were in the intensive care unit (ICU) on the third PO day, while they were in the ward on the sixth PO day; thus, the time interval between evaluations is justified because the clinical presentation of the patients in the two postoperative periods is different with regard to functional performance.

PO - postoperative; RMS - respiratory muscle strength; PMS - peripheral muscle strength.

For the evaluation of RMS, the analog manovacuometer M120 (Porto Alegre, RS, Brazil) was used, with a scale of ± 120cmH_2_O. Respiratory muscle strength tests were performed with patients seated, lower limbs hanging, and feet supported. The nostrils were occluded with a nasal clip and the mouthpiece of the equipment was coupled to the mouth. To evaluate the maximal expiratory pressure (EP_max_), the patient took a deep inspiration to total lung capacity (TLC), during which the occlusion of the buccal orifice was performed, followed by a maximal expiration until the residual volume was sustained for at least 2 seconds. To assess maximal inspiratory pressure (IP_max_), an expiration to residual volume was taken, followed by occlusion of the orifice and maximal inspiration to TLC; the patient then maintained sustained strength for at least 2 seconds. Both maneuvers were repeated three times with a one-minute interval, and the best measure was recorded for statistical analysis. Differences of 10% or less between values were accepted for each repetition. For the calculation of the predicted pressures, the equations proposed by Neder et al.^(16)^ were used.

For the measurement of PMS, the Medical Research Council (MRC) scale was used. This is a commonly used scale that is easy to execute and is low-cost. The individuals remained seated in a chair with the hip joint at 90º of flexion, the knee at 60º of flexion, and the trunk erect. The MRC test measures muscle strength capable of joint displacement against manual resistance applied by the evaluator during the following joint movements: shoulder abduction, elbow flexion, wrist extension, hip flexion, knee extension, and ankle dorsiflexion. When the scores for each evaluated movement are summed, the final MRC score ranges from 0 (tetraplegia) to 60 (normal muscle strength); patients with scores lower than 48 are considered to have muscle weakness.^(17)^ Measurements were repeated three times, with a one-minute interval, and the best measure was recorded for statistical analysis.

Pain intensity was obtained by the visual analog scale (VAS), which consists of a horizontal line 10cm in length that shows a range of pain levels from absence of pain to the most intense pain and (provides a simple and efficient pain intensity measurement.^(18)^

### Statistical analysis

The data were entered into and stored in a database created in Microsoft Excel 2010 software (Redmond, WA, USA). Continuous variables were presented as the mean and standard deviation; categorical variables were presented as relative and absolute frequencies. Normality was tested using the Shapiro-Wilk test. Changes over time in respiratory and peripheral muscle forces were compared using the Kruskal-Wallis test. Correlations were evaluated using the Spearman correlation coefficient. An alpha value of 5% was adopted, and the Statistical Package for Social Science (SPSS) version 20.0 (IBM Inc., Chicago, IL, USA) and BioStat^®^ 5.3 (Belém, PA, Brazil) were used.

Cohen's d was used to determine the clinical effect size of the proposed physiotherapeutic interventions, with the interpretation based on the classification established by Cohen^(19)^ and Fernández-Lao et al.:^(20)^ less than 0.20, negligible effect; 0.20 to 0.50, small effect; 0.50 to 0.80, moderate effect; and > 0.80, large effect.

## RESULTS

Forty-six patients were enrolled and evaluated in the study, with no losses. Thus, 17 (36.96%) women and 29 men (63.04%) with a mean age of 60.50 years (standard deviation [SD] = 9.20) and a mean body mass index (BMI) of 26.60kg/m^2^ (SD = 4.40) were included. Thirty-six (78.26%) patients had systemic arterial hypertension, and 21 (45.65%) had type 2 diabetes mellitus, whereas 22 (47.83%) patients never smoked, 14 (30.43%) had not smoked for at least 6 months, and 10 (21.74%) were smokers.

Regarding the surgeries, MRS was performed in 36 (78.26%) patients, replacement of the aortic valve in 5 (10.87%) patients, and replacement of the mitral valve in 5 (10.87%) patients. Of these, 41 (89.13%) surgeries were performed with extracorporeal circulation, with a mean time of 69.10 minutes (SD = 38.70). The mean length of hospital stay was 7.10 days (SD = 2.1) (Table 1). Regarding the frequency of postoperative complications, only one patient had a pleural effusion, and two patients had pulmonary hypersecretion.

**Table 1:** Clinical, demographic and surgical data for the study patients

Variables	Results
Demographics and anthropometrics	
Age (years)	60.5 (9.2)
Weight (kg)	74.7 (14.0)
Height (m)	1.66 (0.07)
BMI (kg/m²)	26.6 (4.4)
ECC time (min)	69.1 (38.7)
Sex	
Male	29 (63)
Female	17 (37)
Types of surgery	
Myocardial revascularization	36 (78.3)
Replacement of the mitral valve	5 (10.9)
Replacement of the aortic valve	5 (10.9)
Cardiovascular risks	
Smoker	
No	22 (47.8)
Ex-smoker	14 (30.4)
Yes	10 (21.7)
Systemic arterial hypertension	
Yes	36 (78.3)
No	10 (21.7)
Diabetes mellitus	
Yes	21 (45.7)
No	25 (54.3)
Dyslipidemia	
Yes	14 (30.4)
No	32 (69.6)

BMI - body mass index; ECC - extracorporeal circulation. The results are presented as mean (SD) or n (%).

As shown in table 2, there was a significant reduction of RMS and PMS and a significant increase in pain intensity on the third and sixth PO day(p < 0.05), except for the variable IP_max_, which on the sixth PO day already had values similar to the presurgical value and predicted (p > 0.05).

**Table 2:** Comparison of respiratory and peripheral muscle strength and pain intensity over time

Variables	Predicted	Presurgery	3rd PO day	6th PO day
IP_max_ (cmH_2_O)	-102.50 (-82.90 - -109.30)	-120.00 (-85.00 - -120.00)	-80.00 (-40.00 - -120.00)^*.†^	-120.00 (-55.00 - -120.00)
EP_max_ (cmH_2_O)	111.84 (82.60 - 119.13)	90.00 (60.00 - 115.00)*	60.00 (40.00 - 80.00)^*.†^	50.00 (47.50 - 85.00)^*.†^
MRC (score)		60.00 (54.50 - 60.00)	51.00 (46.50 - 56.00)†	55.00 (48.00 - 58.00)†
VAS (score)		0 (0 - 0)	2.00 (0 - 6)†	2.00 (0 - 3.50)†

PO - postoperative; IP_max_ - maximum inspiratory pressure; EP_max_ - maximum expiratory pressure; MRC - Medical Research Council scale; VAS - Visual Analog Scale.

* Differs from the predicted (p < 0.05, Kruskal-Wallis Test post hoc Dunn)

† Differs from presurgery (p < 0.05, Kruskal-Wallis Test post hoc Dunn).

Curiously, there was a positive association between EPmax and PMS at different time points. Pain intensity was not correlated with RMS or PMS. Further details are described in table 3.

**Table 3:** Correlation between respiratory and peripheral muscle strength and pain intensity

Correlation	Presurgery	3rd PO day	6th PO day
IP_max_*versus* EP_max_	r_s_ = 0.397. p = 0.006*	r_s_ = 0.675. p = 0.000*	r_s_ = 0.598. p = 0.000*
IP_max_*versus* MRC	r_s_ = 0.115. p = 0.447	r_s_ = -0.125. p =0.406	r_s_ = 0.289. p = 0.055
IP_max_*versus* VAS	r_s_ = -0.275. p = 0.064	r_s_ = -0.274. p = 0.066	r_s_ = -0.244. p = 0.106
EP_max_*versus* MRC	r_s_ = 0.383. p = 0.009*	r_s_ = 0.468. p = 0.001*	r_s_ = 0.311. p = 0.037*
EP_max_*versus* VAS	r_s_ = -0.174. p = 0.246	r_s_ = -0.086. p = 0.571	r_s_ = -0.190. p = 0.211
MRC *versus* VAS	r_s_ = 0.024. p = 0.872	r_s_ = -0.219. p = 0.143	r_s_ = -0.183. p = 0.223

POD - postoperative; IP_max_ - maximum inspiratory pressure; EP_max_ - maximum expiratory pressure; MRC - Medical Research Council scale; VAS - Visual Analog Scale.

* Significant correlation (p ≤ 0.05, Spearman correlation coefficient).

Table 4 shows the consistently moderate-to-large effect sizes for RMS, MRC and VAS, particularly between the preoperative assessment and the sixth PO day.

**Table 4:** Clinical effect sizes for measurements at pre- and postoperative days

Variables	Cohen’s d
IP_max_	
Pre *versus* 3º PO day	0.60*
Pre *versus* 6º PO day	0.24
3º POD *versus* 6º PO day	-0.34
EP_max_	
Pre *versus* 3º PO day	0.86†
Pre *versus* 6º PO day	0.66*
3º POD *versus* 6º PO day	0.66*
MRC	
Pre *versus* 3º PO day	1.18†
Pre *versus* 6º PO day	0.67*
3º POD *versus* 6º PO day	-0.52*
VAS	
Pre *versus* 3º PO day	-1.17†
Pre *versus* 6º PO day	-0.98†
3º POD *versus* 6º PO day	0.22

IP_max_ - maximum inspiratory pressure; PO - postoperative day; EP_max_ - maximum expiratory pressure; MRC - Medical Research Council scale; VAS - Visual Analog Scale.

* Moderate effect size

† large effect size.

## DISCUSSION

The main results of the present study were as follows: (1) EPmax was reduced at all time points compared to predicted, whereas IPmax returned to preoperative values on the sixth PO day; (2) PMS was reduced after the surgery; (3) postoperative pain intensity increased through at least the sixth PO day; (4) RMS was directly related to PMS; and (5) RMS was not correlated with postoperative pain intensity.

Although certain patient- and cardiac surgery-related risk factors are not modifiable, knowledge of these factors is important for the health team, allowing them to focus increased attention on the patients at greater risk to prevent complications, morbidity, and death.^(21,22)^

In the present study, there was a significant decrease in RMS seen on the third PO day; however, it had returned at the sixth PO day. Corroborating in part the results of the current study, Roncada et al.^(23)^ concluded that after coronary artery bypass grafting surgery, there was a major reduction in pulmonary function, which the author attributed to changes in circulatory factors that affect the synthesis of muscle proteins. In contrast, Urell et al.^(9)^ observed that RMS was not reduced at two months after cardiac surgery. However, the authors did not evaluate patients in the immediate postoperative period; this may be particularly relevant because pain would have needed to be considered.

In an evaluation of the clinical efficacy and feasibility of a respiratory muscular training device applied to patients after cardiothoracic surgery, Crisafulli et al.^(24)^ observed improvement in both IP_max_ and EP_max_ 14 days after surgical procedures associated with the use of the device. While IP_max_ refers mainly to the force of the diaphragm, EP_max_ mainly reflects the strength of the abdominal and intercostal muscles.^(25)^ In the present study, the reduction in IP_max_ was less than the reduction in EP_max_. This could be explained by the fact that peak postoperative diaphragmatic dysfunction (decreased IP_max_) occurs between two and eight hours after surgery, whereas the muscles associated with EP_max_ suffer greater damage from incision and surgical manipulation.^(26)^

Saglam et al.^(27)^, Faustini Pereira et al.^(28)^ and Santos et al.^(7)^ reported that the loss of RMS was related to the decline in PMS, corroborating the results of the present study, which demonstrated a positive and significant correlation between EPmax and PMS evaluated by MRC both in the preoperative period and at both POD time points, that is, the third and sixth PO day. Santos et al.,^(7)^ Saglam et al.,^(27)^ Faustini Pereira et al.,^(28)^ also reported that peripheral muscle strength showed an initial postsurgical loss with partial recovery during the PO period. This result is inconsistent with the results of the present study. However, it should be noted that the postsurgical limitations may last for a period of six weeks to six months.^(7)^ In addition, it is already shown in the literature that worsening of RMS and PMS, as well as cognitive and motor disorders, can be caused by neuromuscular lesions from factors such as mechanical ventilation,^(29,30)^ anesthesia, extracorporeal circulation time,^(31)^ medicines,^(26)^ malnutrition,^(32)^ and bed immobility. Additionally, a study conducted by Santos et al., which evaluated the PMS of patients undergoing elective cardiac surgery, concluded that PMS values were remarkably reduced after surgery and returned to near baseline by the time of discharge,^(7)^ corroborating the results of the present study.

In recent years, protocols for the early withdrawal of sedation and for early mobilization have been used in several intensive centers.^(11,33,34)^ Such protocols highlight the importance physiotherapeutic intervention, which facilitates important gains in both RMS and PMS and is a viable and safe strategy that prevents complications, reduces the deleterious effects of immobility, preserves muscle strength, and thus results in higher functional performance.^(12,35,36)^

The decrease in RMS and PMS in the postoperative period seems to maintain a direct relationship with pain.^(37)^ In the current study, there was no correlation between VAS and RMS. The study of Sasseron et al.,^(38)^ which aimed to evaluate the intensity and location of pain during hospitalization and its effects on the RMS of cardiac surgery patients, showed a correlation between pain in the first PO day and the decrease in the IP_max_. Decreased PMS in individuals with heart disease has been reported in the literature. In addition, peripheral muscle weakness is associated with reduced muscle strength and loss of physical function.^39^ Another study with the objective of evaluating the interaction between handgrip strength (HGS) and myocardial oxygen consumption index (MVO_2_) before and after cardiac surgery showed that hand grip strength had different effects on MVO_2_ prior to and after myocardial revascularization; HGS might be used as a predictor to assess oxygen consumption in cardiac patients.^(40)^

In our study, there was a significant increase in pain on the third PO day, with maintenance of the pain intensity until the sixth PO day, corroborating the findings of Andrade et al.^(41)^ who observed increased pain until the fourth PO day. Pain intensity was considered tolerable (VAS between 2 and 3). Similarly, in previous studies,^(14,42)^ patients most frequently mentioned the surgical incision in the sternal region as the site of pain.

Interestingly, although pain evaluated by the VAS did not show a significant difference, a moderate to high clinical difference was observed over the third and sixth PO day. Pain could have been a limiting factor for RMS; however, that was not observed in this study. Corroborating our study, Urell et al. showed that RMS was not impaired, neither before nor two months after cardiac surgery.^(9)^

The findings indicate that evaluation and monitoring of RMS and PMS is indispensable and assists in the analysis of severity, assessment of functional implications, and determination of the risks of pulmonary and neuromuscular dysfunction, thus providing information supporting the need for adequate muscular training to improve the strength of the respiratory and peripheral muscles and thus improving functional capacity.

The present study has some limitations. First, the number of patients was relatively small. Second, there is no gold standard for the assessment of PMS; however, dynamometry has been demonstrated in the literature to be an effective, practical and reproducible method. In this respect, we affirm that all measures were carried out by strictly following the guidelines to ensure the adequacy and standardization of the test procedures. Finally, we understand that limiting upper limb movements may have biased MRC responses, although the assessment of peripheral muscle strength was tested in small ranges of motion.

## CONCLUSION

We conclude that there is a decrease in respiratory and peripheral muscle strength associated with cardiac surgery. In addition, maximal expiratory pressure is the variable that is most associated with peripheral muscle strength. Thus, to improve both respiratory and peripheral muscle strength, professionals working in intensive care settings should consider these variables in relation to preoperative and postoperative interventions.

## References

[1] Peric V, Stolic R, Jovanovic A, Grbic R, Lazic B, Sovtic S (2017). Predictors of quality of life improvement after 2 years of
coronary artery bypass surgery. Ann Thorac Cardiovasc Surg.

[2] Hawkes AL, Nowak M, Bidstrup B, Speare R (2006). Outcomes of coronary artery bypass graft surgery. Vasc Health Risk Manag.

[3] Siregar S, Groenwold RH, de Mol BA, Speekenbrink RG, Versteegh MI, Brandon Bravo Bruinsma GJ (2013). Evaluation of cardiac surgery mortality rates 30-day mortality or
longer follow-up?. Eur J Cardiothorac Surg.

[4] Scherner M, Madershahian N, Kuhr K, Rosenkranz S, Stöger E, Rahmanian P (2014). Aortic valve replacement after previous heart surgery in
high-risk patients transapical aortic valve implantation versus conventional
aortic valve replacement-a risk-adjusted and propensity score-based
analysis. J Thorac Cardiovasc Surg.

[5] van Venrooij LM, Verberne HJ, de Vos R, Borgmeijer-Hoelen MM, van Leeuwen PA, de Mol BA (2012). Postoperative loss of skeletal muscle mass, complications and
quality of life in patients undergoing cardiac surgery. Nutrition.

[6] Simsek T, Simsek HU, Cantürk NZ (2014). Response to trauma and metabolic changes posttraumatic
metabolism. Ulus Cerrahi Derg.

[7] Santos KM, Cerqueira Neto ML, Carvalho VO, Santana Filho VJ, Silva Junior WM, Araújo Filho AA (2014). Evaluation of peripheral muscle strength of patients undergoing
elective cardiac surgery a longitudinal study. Rev Bras Cir Cardiovasc.

[8] Iida Y, Yamazaki T, Kawabe T, Usui A, Yamada S (2014). Postoperative muscle proteolysis affects systemic muscle weakness
in patients undergoing cardiac surgery. Int J Cardiol.

[9] Urell C, Emtner M, Hedenstrom H, Westerdahl E (2016). Respiratory muscle strength is not decreased in patients
undergoing cardiac surgery. J Cardiothorac Surg.

[10] Hermes BM, Cardoso DM, Gomes TJ, Santos TD, Vicente MS, Pereira SN (2015). Short-term inspiratory muscle training potentiates the benefits
of aerobic and resistance training in patients undergoing CABG in phase II
cardiac rehabilitation program. Rev Bras Cir Cardiovasc.

[11] da Costa Torres D., Dos Santos PR., Reis HJ., Paisani DM., Chiavegato LD (2016). Effectiveness of an early mobilization program on functional
capacity after coronary artery bypass surgery A randomized controlled trial
protocol. SAGE Open Med.

[12] Cordeiro AL, de Melo TA, Neves D, Luna J, Esquivel MS, Guimarães AR (2016). Inspiratory muscle training and functional capacity in patients
undergoing cardiac surgery. Braz J Cardiovasc Surg.

[13] Caruso FR, Arena R, Phillips SA, Bonjorno Jr JC, Mendes RG, Arakelian VM (2015). Resistance exercise training improves heart rate variability and
muscle performance a randomized controlled trial in coronary artery disease
patients. Eur J Phys Rehabil Med.

[14] Sattari M, Baghdadchi ME, Kheyri M, Khakzadi H, Ozar Mashayekhi S (2013). Study of patient pain management after heart
surgery. Adv Pharm Bull.

[15] Mello LC, Rosatti SF, Hortense P (2014). Assessment of pain during rest and during activities in the
postoperative period of cardiac surgery. Rev Lat Am Enfermagem.

[16] Neder JA, Andreoni S, Lerario MC, Nery LE (1999). Reference values for lung function tests II. Maximal respiratory
pressures and voluntary ventilation. Braz J Med Biol Res.

[17] Paternostro-Sluga T, Grim-Stieger M, Posch M, Schuhfried O, Vacariu G, Mittermaier C (2008). Reliability and validity of the Medical Research Council (MRC)
scale and a modified scale for testing muscle strength in patients with
radial palsy. J Rehabil Med.

[18] Ferreira-Valente MA, Pais-Ribeiro JL, Jensen MP (2011). Validity of four pain intensity rating scales. Pain.

[19] Cohen J (1977). Statistical power analysis for the behavioral sciences.

[20] Fernández-Lao C, Cantarero-Villanueva I, Fernández-de-las-Peñas C, del Moral-Ávila R, Castro-Sánchez AM, Arroyo-Morales M Effectiveness of a multidimensional physical therapy program on
pain, pressure hypersensitivity, and trigger points in breast cancer
survivors: a randomized controlled clinical trial. Clin J Pain.

[21] Platz JJ, Fabricant L, Norotsky M (2017). Thoracic trauma onjuries, evaluation, and
treatment. Surg Clin North Am.

[22] Stolinski J, Plicner D, Fijorek K, Grudzien G, Kruszec P, Andres J (2017). Respiratory system function in patients after aortic valve
replacement through right anterior minithoracotomy. Thorac Cardiovasc Surg.

[23] Roncada G, Dendale P, Linsen L, Hendrikx M, Hansen D (2015). Reduction in pulmonary function after CABG surgery is related to
postoperative inflammation and hypercortisolemia. Int J Clin Exp Med.

[24] Crisafulli E, Venturelli E, Siscaro G, Florini F, Papetti A, Lugli D (2013). Respiratory muscle training in patients recovering recent open
cardiothoracic surgery a randomized-controlled trial. Biomed Res Int.

[25] Sachs MC, Enright PL, Hinckley Stukovsky KD, Jiang R, Barr RG, Multi-Ethnic Study of Atherosclerosis Lung Study the M-ES of A
L (2009). Performance of maximum inspiratory pressure tests and maximum
inspiratory pressure reference equations for 4 race/ethnic
groups. Respir Care.

[26] Sasaki N, Meyer MJ, Eikermann M (2013). Postoperative respiratory muscle dysfunction pathophysiology and
preventive strategies. Anesthesiology.

[27] Saglam M, Vardar-Yagli N, Calik-Kutukcu E, Arikan H, Savci S, Inal-Ince D (2015). Functional exercise capacity, physical activity, and respiratory
and peripheral muscle strength in pulmonary hypertension according to
disease severity. J Phys Ther Sci.

[28] Faustini Pereira JL, Galant LH, Rossi D, Telles da Rosa LH.Garcia E.de Mello Brandão AB (2016). Functional capacity, respiratory muscle strength, and oxygen
consumption predict mortality in patients with cirrhosis. Can J Gastroenterol Hepatol.

[29] Tobin MJ, Laghi F, Jubran A (2010). Narrative review ventilator-induced respiratory muscle
weakness. Ann Intern Med.

[30] Baldwin CE, Bersten AD (2014). Alterations in respiratory and limb muscle strength and size in
patients with sepsis who are mechanically ventilated. Phys Ther.

[31] Calles AC, Lira JL, Granja KS, Medeiro JD, Farias AR, Cavalcanti RC (2016). Pulmonary complications in patients undergoing coronary artery
bypass grafting at a hospital in Maceio, Brazil. Fisioter Mov.

[32] Dassios T, Katelari A, Doudounakis S, Dimitriou G (2013). Aerobic exercise and respiratory muscle strength in patients with
cystic fibrosis. Respir Med.

[33] Cassina T, Putzu A, Santambrogio L, Villa M, Licker MJ (2016). Hemodynamic challenge to early mobilization after cardiac surgery
a pilot study. Ann Card Anaesth.

[34] Ramos dos Santos PM.Aquaroni Ricci N.Aparecida Bordignon Suster
É.de Moraes Paisani D.Dias Chiavegato L (2017). Effects of early mobilisation in patients after cardiac surgery a
systematic review. Physiotherapy.

[35] Urell C, Westerdahl E, Hedenström H, Janson C, Emtner M (2012). Lung function before and two days after open-heart
surgery. Crit Care Res Pract.

[36] Urell C. (2013). Lung function, respiratory muscle strength and effects of
breathing exercises in cardiac surgery patients. Acta Universitatis Upsaliensis.

[37] Westerdahl E, Jonsson M, Emtner M (2016). Pulmonary function and health-related quality of life 1-year
follow up after cardiac surgery. J Cardiothorac Surg.

[38] Sasseron AB, Figueiredo LC, Trova K, Cardoso AL, Lima NM, Olmos SC (2009). Does the pain disturb the respiratory function after open heart
surgery. Rev Bras Cir Cardiovasc.

[39] Norman K, Stobäus N, Gonzalez MC, Schulzke JD, Pirlich M (2011). Hand grip strength outcome predictor and marker of nutritional
status. Clin Nutr.

[40] Sokran SN, Mohan V, Kamaruddin K, Sulaiman MD, Awang Y, Othman IR (2015). Hand grip strength and myocardial oxygen consumption index among
coronary artery bypass grafting patients. Iran J Med Sci.

[41] Andrade ÉV, Barbosa MH, Barichello E (2010). Avaliação da dor em pós-operatório de
cirurgia cardíaca. Acta Paul Enferm.

[42] Bigeleisen PE, Goehner N (2015). Novel approaches in pain management in cardiac
surgery. Curr Opin Anaesthesiol.

